# Supplementary material for: The Nutritional, ACE Inhibition, and Antioxidant Properties of Hydrolysate Powders Derived from Different Stages of Thai Silkworm (Bombyx mori)
Source: Foods. 2025 Nov 23;14(23):4018. doi: 10.3390/foods14234018 (PMC12691913; doi:10.3390/foods14234018)
Supplement: Supplementary file 1 [file foods-14-04018-s001.zip › foods-3981320-supplementary.pdf]

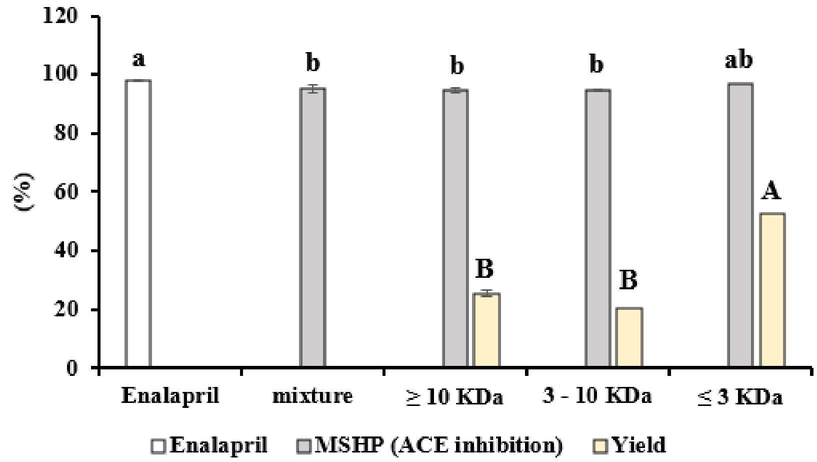

Figure S1 Comparison of ACE-inhibitory activity and fraction yield of mature silkworm hydrolysate powder (MSHP) across different molecular-weight ranges. Data are presented as mean ± standard deviation (SD, n = 3). Statistical differences were evaluated by one-way ANOVA followed by Duncan's multiple range test ($p < 0.05$). Values with different superscript lowercase letters (a–b) differ significantly in ACE-inhibitory activity, while values with different superscript uppercase letters (A–B) differ significantly in yield.
